# Early Post-Natal Immune Activation Leads to Object Memory Deficits in Female *Tsc2*^+/−^ Mice: The Importance of Including Both Sexes in Neuroscience Research

**DOI:** 10.3390/biomedicines12010203

**Published:** 2024-01-17

**Authors:** Manuel F. López-Aranda, Karen Bach, Raymond Bui, Miranda Phan, Odilia Lu, Chirag Thadani, Alessandro Luchetti, Rochelle Mandanas, Isaiah Herrera, María Dolores López-Ávalos, Alcino J. Silva

**Affiliations:** 1 Departamento de Biología Celular, Genética y Fisiología, Facultad de Ciencias, Universidad de Málaga, 29010 Málaga, Spain; 2 Departments of Neurobiology, Psychology, Psychiatry, Integrative Center for Learning and Memory and Brain Research Institute, University of California Los Angeles, Los Angeles, CA 90095, USAsilvaa@g.ucla.edu (A.J.S.); 3 Instituto de Investigación Biomédica de Málaga (IBIMA)-Plataforma BIONAND, 29590 Málaga, Spain

**Keywords:** *Tsc2*, female, immune activation, mTOR

## Abstract

There is evidence that viral infections during pre-natal development constitute a risk factor for neuropsychiatric disorders and lead to learning and memory deficits. However, little is known about why viral infections during early post-natal development have a different impact on learning and memory depending on the sex of the subject. We previously showed that early post-natal immune activation induces hippocampal-dependent social memory deficits in a male, but not in a female, mouse model of tuberous sclerosis complex (TSC; *Tsc2*^+/−^ mice). Here, we explored the impact of a viral-like immune challenge in object memory. We demonstrate that early post-natal immune activation (during the first 2 weeks of life) leads to object memory deficits in female, but not male, mice that are heterozygous for a gene responsible for tuberous sclerosis complex (*Tsc2*^+/−^ mice), while no effect was observed in wild type (WT) mice. Moreover, we found that the same immune activation in *Tsc2*^+/−^ adult mice was not able to cause object memory deficits in females, which suggests that the early post-natal development stage constitutes a critical window for the effects of immune challenge on adult memory. Also, our results suggest that mTOR plays a critical role in the observed deficit in object memory in female *Tsc2*^+/−^ mice. These results, together with previous results published by our laboratory, showing sex-specific memory deficits due to early post-natal immune activation, reinforce the necessity of using both males and females for research studies. This is especially true for studies related to immune activation, since the higher levels of estrogens in females are known to affect inflammation and to provide neuroprotection.

## 1. Introduction

Evidence shows that viral infections during pre-natal development constitute a risk factor for neuropsychiatric disorders and lead to learning and memory deficits [1,2,3,4,5,6,7,8,9,10,11,12,13,14,15]. However, because most of those studies were carried out using almost exclusively male rodents, little is known about the impact of sex on the learning and memory effects of viral infections during early post-natal development [16,17,18].

Tuberous sclerosis complex (TSC) is an autosomal dominant disorder caused by mutations in either the *TSC2* gene located on chromosome 16p13.3 and encoding tuberin, or the *TSC1* gene located on chromosome 9q34 and encoding hamartin proteins [19,20,21]. TSC is the second most common neurocutaneous disorder (1 in 6000 people are affected worldwide), and is characterized by the growth of numerous benign tumors in many parts of the body, including the brain, skin, heart, lungs and kidneys [22]. Additionally, most TSC patients are affected by neuropsychiatric disorders [23].

Recent publications [24] from our laboratory showed that the challenge of an immune activation in the early post-natal period induces hippocampal-dependent social memory deficits in male, but not female, *Tsc2*^+/−^ mice due to the abnormal mammalian target of rapamycin (mTOR)-dependent interferon signaling and subsequent impairments in microglia function. Interestingly, those male *Tsc2*^+/−^ mice showed no deficits in object memory despite showing impairment in social memory. Here, we evaluate the effects of early post-natal immune activation on object memory of *Tsc2*^+/−^ mice, using the Novel Object Recognition (NOR) task. The NOR test is a cortical-dependent behavioral assay that relies on the animal’s innate preference for novelty and is used to investigate non-spatial object memory. This task measures the animal’s ability to distinguish a novel object from a familiar one [25,26,27,28,29,30].

## 2. Materials and Methods

### 2.1. Experimental Design and Subject Details

To generate the *Tsc2*^+/−^ mice used in this study to evaluate the impact of early post-natal immune activation in object memory of those mice, we crossed male *Tsc2*^+/−^ mice [31] with C57BL/6J wild type (WT) females (JAX, Cat.#: 000664). *Tsc2*^+/−^ male breeders were of a C57BL/6Ncrl genetic background (Charles River Laboratories, Cat.#: 027). The pregnant female mice were individually housed and not disturbed, except for a weekly cage change. The pregnancy was confirmed by checking for abdominal distension. Daily checks were conducted on the pregnant females to determine the exact day of parturition (designated as P0). To activate the immune system early post-natally, the pups were intraperitoneally injected with either 20 mg/kg Poly I:C or a vehicle on P3, P7 and P14. To assess the impact of immune activation in adult *Tsc2*^+/−^ mice, adult wild-type (WT) and mutant mice (*Tsc2*^+/−^) were injected with 20 mg/kg Poly I:C three times, followed by a similar schedule to that used during early post-natal development. Tail biopsies for genotyping were collected around P40.

### 2.2. Poly I:C Administration

The Poly I:C potassium salt (Sigma; Cat.#: P9582-50MG) was freshly dissolved in vehicle solution (0.9% sterile saline) before each use. It should be noted that Poly I:C is supplied at 10% of the total weight of the salt, and the dosage was based on the weight of Poly I:C itself.

(a) Early post-natal administration: At the age of P3, P7 and P14, mutant *Tsc2*^+/−^ mice and their WT littermates were injected intraperitoneally with 20 mg/kg Poly I:C. At the same time, mice for the control group were injected with the vehicle.
(b) Administration in adults: Adult (4–6 months) *Tsc2*^+/−^ mice and WT littermates were fed with PLX5622 or control chow for 21 days (see below). The mice were injected intraperitoneally with Poly I:C (20 mg/kg) 10 h, 4 days and 11 days after ending the treatment with PLX5622 or control chow. Thus, the schedule of Poly I:C injections overlapped with the period of microglial repopulation after PLX5622 treatment.

### 2.3. Novel Object Recognition (NOR) Test

Adults (4–6 months) *Tsc2*^+/−^ mice and WT littermates were tested using NOR.

The Novel Object Recognition (NOR) test was carried out as described previously [24,32]. This behavioral test involves several steps to assess object memory in mice. For the first step, the mice were handled for eight minutes daily for six consecutive days to acclimate them to the experimenter. Subsequently, over the next two days, they were habituated in an open field (41.5 × 41.5 × 40.5 cm) for 12 min each day. During the training session, the mice were placed in the open field with two identical objects and were allowed to explore freely for seven minutes. After 24 h, the mice were tested for object recognition memory with both a previously presented object and a new object. To avoid any kind of bias during the test, the new object’s location was counterbalanced between trials. The mice were trained or tested only once per day, and the open field was cleaned after each session using 70% ethanol. The sessions were video recorded, and 1–2 blinded and experienced observers scored the object exploration time offline using stopwatches. The variation between observers was normally less than 2 s for the training and test sessions. Only when the mouse touched any of the objects with its nose, was exploration counted. All the stages of the experiments as well as the scoring were carried out blind to genotype and treatment condition. Small bottles and containers of different shapes were the objects used in this study.

### 2.4. PLX5622 Treatment

Plexxikon Inc. provided the PLX6622 and control rodent diet. Research Diets Inc. formulated that rodent diet in in AIN-76A standard chow. Over a period of 21 days, adult *Tsc2*^+/−^ mice, aged 4–6 months, were fed with PLX5622 (1200 mg/kg; Cat.#: D11100404i) or control chow (Cat.#: D10001i) [24,33], with each mouse receiving 5 g of chow per day.

### 2.5. Rapamycin Treatment

We freshly dissolved rapamycin (5 mg/kg; LC Laboratories, Cat.#: R-5000) in DMSO (Sigma-Aldrich, Cat.#: D5879-500ML) before use. Mice injected at an early post-natal age with Poly I:C were, as adults (4–6 months), treated with a daily intraperitoneal injection of rapamycin (5 mg/kg) or vehicle (DMSO) for 5 days prior to the Novel Object Recognition test. Mice were tested for object memory 18 h after the last injection of rapamycin or DMSO.

### 2.6. Immunohistochemistry

This methodology was carried out in accordance with a previously established protocol [34]. To begin, mice were transcardially perfused with a fixative containing 4% paraformaldehyde, and their brains were subsequently cryoprotected with 30% sucrose. Following this, sagittal free-floating brain sections, each 60 μm in thickness, were incubated overnight at 4 °C with polyclonal rabbit anti-Iba1 (Wako Chemicals, Cat.#: 019-19741) at a 1:1000 dilution. Subsequently, the sections were incubated for 90 min with Alexa Fluor 488 goat anti-rabbit IgG (Invitrogen, Cat.#: A11011) at a 1:500 dilution. This was followed by a 15 min incubation in DAPI at a 1:1000 dilution, and a 14 min wash in PBS. The immunofluorescence labeling was then detected using a confocal microscope.

### 2.7. Statistical Analysis

The mouse behavioral data are presented as the mean ± SEM and as individual data. Regarding the behavioral experiments, the statistics presented in the figures were initially based on Student’s *t*-test. Additionally, the data were subjected to analysis using two-way ANOVA plus Holm–Sidak post hoc analyses, and the results obtained were found to be identical. The figure legends provide the *p*, *n* and *t* values, with a significance level of *p* < 0.05 considered significant. *p* values ≥ 0.05 were denoted as non-significant (n.s.), while different levels of significance were indicated as *, **, *** or ****, corresponding to *p* < 0.05, *p* < 0.01, *p* < 0.001 and *p* < 0.0001, respectively. The statistical analyses and the generation of graphical representations of the data in this manuscript were performed using GraphPad Prism 7 software.

## 3. Results

### 3.1. Early Post-Natal Immune Activation Induces Long-Lasting Object Memory Deficits in Adult Tsc2^+/−^ Female Mice

**Figure 1:**
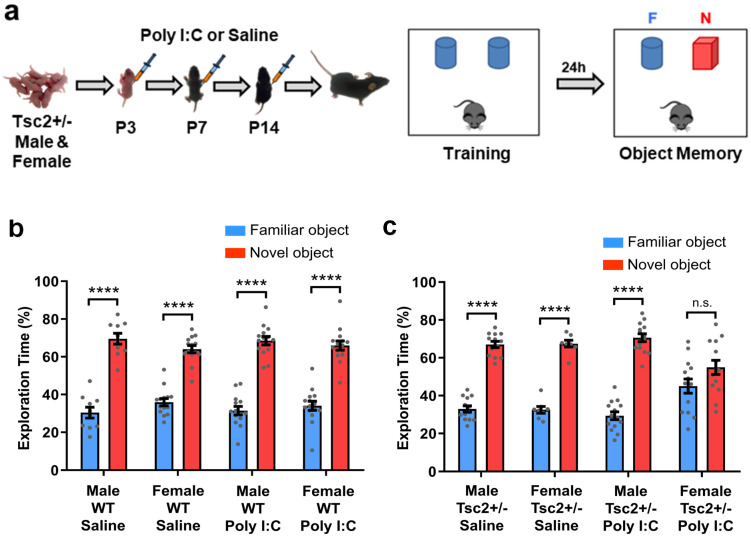
Early post-natal immune activation triggers object memory deficits in female *Tsc2*^+/−^ mice. (**a**) Timeline of injections of polyinosinic:polycytidylic acid (Poly I:C) or Saline and behavior approach. (**b**) Graph shows the percentage of time that mice spent actively exploring the novel and the familiar objects. All groups, male WT/Saline (*n* = 10; **** *p* < 0.0001, *t* = 9.64), female WT/Saline (*n* = 14; **** *p* < 0.0001, *t* = 10.28), male WT/Poly I:C (*n* = 14; **** *p* < 0.0001, *t* = 9.50) and female WT/Poly I:C (*n* = 15; **** *p* < 0.0001, *t* = 9.45) showed normal object memory (they explored significantly more the novel object than the familiar object). (**c**) Male *Tsc2*^+/−^/Saline (*n* = 14; **** *p* < 0.0001, *t* = 14.29), female *Tsc2*^+/−^/Saline (*n* = 8; **** *p* < 0.0001, *t* = 14.07) and male *Tsc2*^+/−^/Poly I:C (*n* = 14; **** *p* < 0.0001, *t* = 13.88) showed normal object memory (they explored the novel object significantly more than the familiar object). Conversely, female *Tsc2*^+/−^/Poly I:C (*n* = 14; n.s. *p* = 0.07, *t* = 1.88) showed deficits in object memory (showed no preference for the novel object). Data represent means ± SEM as well as individual data.

Male and female WT and *Tsc2*^+/−^ mice were injected with either polyinosinic:polycytidylic acid (Poly I:C; 20 mg/kg) or saline (control) i.p. at post-natal day 3 (P3), P7 and P14 (Figure 1a). As adults (4–6 months old), those mice were tested using Novel Object Recognition (NOR) [25,26,27,28,29,30]. WT mice injected with Poly I:C showed normal object memory (Figure 1b). However, female, but not male, *Tsc2*^+/−^ mice injected with Poly I:C early post-natally (*Tsc2*^+/−^ Ep) showed object memory deficits (they showed no preference for the novel object vs. the familiar object during the NOR test; Figure 1c). These results suggest that, in the first two weeks of life, *Tsc2*^+/−^ female (but not male) mice are especially sensitive to the impact of immune activation on object memory.

### 3.2. The Administration of a mTOR Inhibitor (Rapamycin) in Adults Can Reverse the Object Memory Deficits of Female Tsc2^+/−^ Ep Mice

The *Tsc2* gene plays a critical role in the regulation of mTOR signaling [35,36,37,38], leading to the upregulation of mTOR signaling in both rodents and humans in cases where *Tsc2* levels are reduced [31,39]. Previous findings have demonstrated that the administration of rapamycin, an mTOR inhibitor, has the ability to reverse multiple phenotypes observed in *Tsc2*^+/−^ mice [40,41,42].

**Figure 2:**
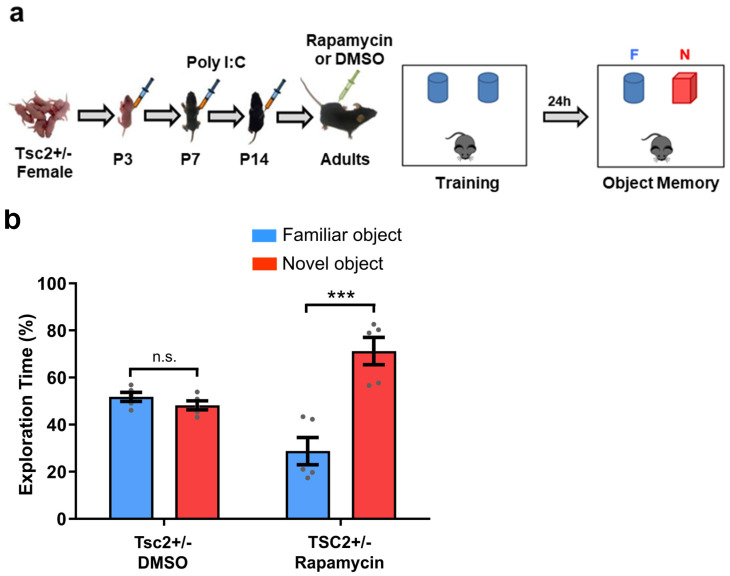
The administration of a mTOR inhibitor (rapamycin) in adults can reverse the object memory deficits of female *Tsc2*^+/−^ Ep mice. (**a**) Timeline of injections of Poly I:C and Rapamycin (or DMSO in control mice) and behavior approach. (**b**) Graph shows the percentage of time that mice spent actively exploring the novel and familiar objects. Female *Tsc2*^+/−^ Ep/DMSO (*n* = 5; n.s. *p* = 0.21, *t* = 1.33) showed deficits in object memory (showed no preference for the novel object). However, female *Tsc2*^+/−^ Ep/Rapamycin (*n* = 5; *** *p* < 0.001, *t* = 5.19) showed normal object memory (they explored the novel object significantly more than the familiar object). Data represent means ± SEM as well as individual data.

Rapamycin was initially developed as an immunosuppressant for organ transplant in humans. Its immunosuppressive effects has been studied in vivo and in vitro [43,44,45]. Rapamycin is also known for its effects as inhibitor of the mTOR signaling pathway [46,47], which integrates intracellular and extracellular signals to regulate the metabolism, growth, proliferation and survival of the cells [48]. Importantly, previous results in our laboratory [24] showed that the administration of rapamycin in male *Tsc2*^+/−^ Ep mice reversed the social memory deficit of these mice. To evaluate whether the inhibition of mTOR signaling in adult female *Tsc2*^+/−^ Ep mice was able to reverse the observed object memory deficits, *Tsc2*^+/−^ Ep mice were treated at an adult age with rapamycin (5 mg/kg) (Figure 2a). Female *Tsc2*^+/−^ Ep mice treated with rapamycin showed normal object memory (preference for the novel object vs. the familiar object; Figure 2b). These results suggest that mTOR signaling has a critical role in the object memory deficits of adult female *Tsc2*^+/−^ Ep mice.

### 3.3. Adult Immune Activation Induces no Deficits in Object Memory in Tsc2^+/−^ Mice

**Figure 3:**
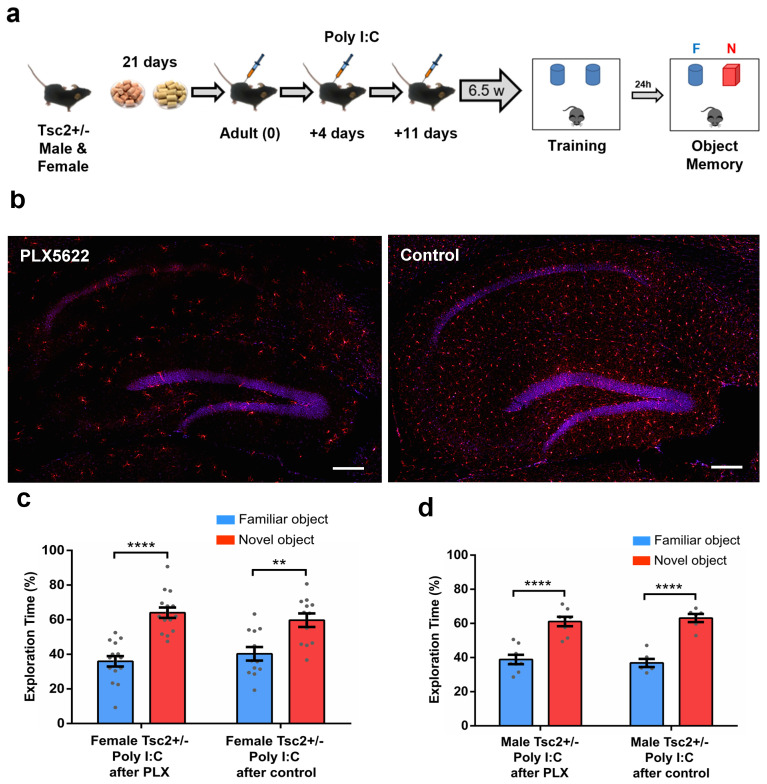
Adult immune activation induces no deficits in object memory in Tsc2^+/−^ mice. (**a**) Timeline for treatment with PLX5622 (PLX), for microglial depletion, or control chow, followed by injections of Poly I:C, and subsequent behavior approach. (**b**) IBA1 immunostaining of *Tsc2*^+/−^ Control and PLX5622-treated mice. Treatment with PLX5622 led to a massive reduction in the microglial population in the whole brain (hippocampus is shown here as example). Bar: 200 µm. (**c**) Female *Tsc2*^+/−^/Poly I:C after PLX (*n* = 15; **** *p* < 0.0001, *t* = 6.66) and female *Tsc2*^+/−^/Poly I:C after control chow (*n* = 12; ** *p* < 0.01, *t* = 3.52) showed normal object memory. (**d**) Male *Tsc2*^+/−^/Poly I:C after PLX (*n* = 8; **** *p* < 0.0001, *t* = 5.73) and male *Tsc2*^+/−^/Poly I:C after control chow (*n* = 6; **** *p* < 0.0001, *t* = 7.76) showed normal object memory. Data represent means ± SEM as well as individual data.

Next, we aimed to evaluate whether the administration of Poly I:C in *Tsc2*^+/−^ adult mice had similar outcomes regarding object memory as we observed in *Tsc2*^+/−^ Ep mice. Immune activation at adult age did not provoke object memory deficits, either in female (Figure 3c) or in male (Figure 3d) *Tsc2*^+/−^ mice.

PLX5622 is a Colony-Stimulating Factor 1 Receptor (CSF1R) inhibitor known for its effects on microglial depletion and its potential therapeutic benefits. Research has shown that the inhibition of the CSF1R results in the almost complete elimination of microglia brain-wide [24,49], followed by repopulation upon inhibitor withdrawal. Thus, such inhibitors represent a valuable tool for assessing the role of this glial cell population. Previous results in our laboratory [24] showed that the administration of Poly I:C in adults *Tsc2*^+/−^ during the repopulation of microglia lead to social memory deficits in both, male and female *Tsc2*^+/−^ mice. Therefore, we used this depletion–repopulation strategy to determine whether the new/immature microglia may re-open a window of sensitivity to immune activation. For this purpose, adult male and female *Tsc2*^+/−^ mice were treated with PLX5622 (a CSF1R inhibitor) or control chow for 21 days. Then, all mice returned to normal chow, and were injected with Poly I:C (20 mg/kg) during the repopulation period (at 0, 4 and 11 days after PLX5622/control chow termination). All mice were tested for object memory at 6.5 weeks after the final Poly I:C injection (Figure 3a).

By sacrificing sample mice after 21 days of PLX5622/control chow, we first confirmed that the administration of PLX5622 led to a considerable depletion of microglia (Figure 3b). Regarding object memory, neither female (Figure 3c) nor male Tsc2^+/−^ mice (Figure 3d) showed any deficits. These results suggest that immune activation in adult Tsc2^+/−^ mice does not induce deficits in object memory (as revealed mice treated with control chow). Moreover, the same immune challenge in *Tsc2*^+/−^ mice in the microglial repopulation/maturation stage (mice treated with PLX5622) yielded similar results in object recognition task, indicating that microglial immaturity does not seem to re-open a vulnerable window to immune activation for object memory. Taken together, these data indicate that during the first two weeks of life, the object memory of *Tsc2*^+/−^ female mice is especially sensitive to immune activation. Also, this sensitivity is sex-dependent, as male mice did not show object memory deficits.

## 4. Discussion

Sex-specific effects, even in animal studies, are often overlooked in neuroscience due to the increased workload necessary to balance male and female subjects. However, the observation of different results in males versus females can be extremely important because these findings help to generate hypotheses about the mechanisms of the observed biological phenomena. Previously [24], our laboratory showed that early post-natal immune activation induces social memory deficits in male, but not female, *Tsc2*^+/−^ mice due to an abnormal mammalian target of rapamycin (mTOR)-dependent interferon signaling and subsequent impairments in microglia function. Interestingly, male *Tsc2*^+/−^ mice showed no deficits in object memory. Here, we report that early post-natal immune activation triggers an object memory deficit in female, but not male, *Tsc2*^+/−^ mice (Table 1). Moreover, the same immune activation applied to adults did not provoke such memory deficits, highlighting the vulnerability of post-natal developmental stages to immune activation. We further showed that treatment with Rapamycin can reverse this object memory deficit, which suggests that mTOR plays an important role in the underlying mechanism. Together, these results strongly suggest that the early post-natal stages of development constitute a vulnerable window where immune activation can trigger learning and memory deficits in *Tsc2*^+/−^ mice. Moreover, it seems that mTOR plays a critical role in both phenotypes, as demonstrated by our finding that Rapamycin treatment can reverse both social [24] and object memory deficits. In fact, hamartin (TSC1) and tuberin (TSC2) protein complex negatively regulates mTOR signaling, so the dysregulation of such complex results in mTOR signaling overactivation [50]. Interestingly, clinical trials targeting mTOR inhibition have been carried out for certain symptoms (subependymal astrocytoma, angiomyolipoma) in tuberous sclerosis (TSC) patients [51,52,53] and is also considered an option for treating neurological symptoms [40,54,55]. In line with this idea, here we showed the reversal of object memory impairment by Rapamycin treatment, which further supports mTOR inhibition as a plausible strategy for treating neurological symptoms in TSC patients.

Remarkably, the same immune challenge taking place at the same stage of development (i.e., post-natally) triggers different memory phenotypes in a sex-dependent manner in *Tsc2*^+/−^ mice. This may be due to the fact that those phenotypes can be associated with two different areas of the brain. The NOR test is a neurobehavioral assay known to be cortical-dependent [56,57], as shown by studies with cortical lesions [58,59,60,61,62], optogenetic stimulation [63] and neuronal activation [64]. On the other hand, social memory has been associated with other brain regions, including the hippocampus [65,66,67].

Our studies of *Tsc2*^+/−^ mice [24], including those presented here, show how early post-natal immune activation leads to social memory (hippocampal-dependent) deficits only in male mice, while the same immune challenge leads to object memory (cortical-dependent) deficits exclusively in female *Tsc2*^+/−^ mice. Thus, we speculate that early post-natal immune activation in *Tsc2*^+/−^ mice leads to different kinds and/or levels of response in the brain region in a sex-dependent manner. Future studies are required to evaluate this hypothesis and to determine the mechanisms underlying these memory deficits in *Tsc2*^+/−^ mice.

TSC is a genetic disorder with an incidence of 1 in 6000 people worldwide caused by mutations in the *TSC1* or *TSC2* genes [23]. Amongst a variety of symptoms, most patients (>90%) are affected by an ample spectrum of cognitive and/or behavioral problems, most of them related to neurodevelopment, including autism spectrum disorders (ASD) (25–50% of TSC cases; [23,68]). Our results support the already described the interactions of early immune activation (e.g., infections in early childhood) within a specific genetic background in neurodevelopment and underline the importance of preventing severe infections during this (early post-natal) developmental stage. Also, they provide further evidence of the sex-dependent differential susceptibility of different neurodevelopmental processes, which may underlie the sex-based bias in those cognitive and behavioral problems.

Historically, neuroscience research has preferentially used male instead of female mice for experiments, with the purpose of avoiding the putative and unknown impact of female cycling hormones on the phenomenon under study. To encourage scientists to use both male and female groups, in 2015 multiple institutions, including the U.S. National Institutes of Health (NIH), established policies to require applicants to report plans to balance male and female samples, cells and animals for their research [69]. Using female mice for research is especially important for neuroprotection studies because estrogen, which is primarily produced in the ovaries, plays an important neuroprotective and anti-inflammatory role [70] in neurological disorders and insults such as stroke, brain injury, Alzheimer’s disease and Parkinson’s disease [71].

This work emphasizes the necessity of considering both males and females for research studies. Clarifying the mechanisms underlying the different phenotypes observed between males and females, in response to the same challenge (such as immune activation), is critical for developing drugs and treatments for those phenotypes.

## Figures and Tables

**Table 1:** Effects of early post-natal immune activation in *Tsc2*^+/−^ mice. Summary of our previous and current results. Early post-natal immune activation induces social memory deficits in male but not female *Tsc2*^+/−^ mice. Here we show that the same immune activation induces object memory deficits in female but not male *Tsc2*^+/−^ mice. √: Normal memory. X: Memory deficits.

Early Post-Natal Immune Activation		Social Memory (Hippocampal Dependent)	Object Memory (Cortical Dependent)
Male	WT	√	√
	*Tsc2* ^+/−^	X	√
Female	WT	√	√
	*Tsc2* ^+/−^	√	X

## Data Availability

PLX5622 was obtained under a material transfer agreement with Plexxikon. All data needed to evaluate the conclusions in the paper are present in the paper.
